# Upregulation of Spinal miR-155-5p Contributes to Mechanical Hyperalgesia by Promoting Inflammatory Activation of Microglia in Bone Cancer Pain Rats

**DOI:** 10.3390/life12091349

**Published:** 2022-08-30

**Authors:** Yanping Jian, Zongbin Song, Zhuofeng Ding, Jian Wang, Ruike Wang, Xinran Hou

**Affiliations:** 1 Department of Anesthesiology, Xiangya Hospital, Central South University, Changsha 410008, China; 2 Department of Anesthesiology, Marine Corps Hospital of PLA, Chaozhou 521000, China

**Keywords:** bone cancer pain, mechanical hyperalgesia, miR-155-5p, *Sgk3*, microglia

## Abstract

Bone cancer pain (BCP) seriously deteriorates the life quality of patients, but its underlying mechanism is still unclear. Spinal microRNAs might contribute to the development of BCP and the role of microglial activation is controversial. In this study, we established a BCP model by injecting Walker 256 breast carcinoma cells into the tibial intramedullary cavity of rats and significant hyperalgesia was observed in the BCP rats. The lumbar spinal cords were harvested to perform RNA sequencing (RNA-seq), and 31 differentially expressed miRNAs (26 upregulated and 5 downregulated) were identified in the BCP rats. Among them, miR-155-5p was significantly upregulated in the BCP rats. Spinal microglial activation was observed during BCP development. miR-155-5p could be expressed in spinal microglia and was significantly upregulated in microglia treated with lipopolysaccharide (LPS) in vitro. Serum/glucocorticoid regulated kinase family member 3 (*Sgk3*) was predicted to be the possible downstream target of miR-155-5p and this was confirmed using a dual-luciferase reporter assay in vitro. The inhibition of miR-155-5p restored *Sgk3*-expression-attenuated microglial activation and alleviated hyperalgesia in the BCP rats. In conclusion, spinal miR-155-5p/*Sgk3*/microglial activation might play an important role in BCP pathogenesis.

## 1. Introduction

Bone cancer pain (BCP) is chronic pain caused by primary or metastatic tumors damaging or injuring the bony skeleton; BCP is the most common type of chronic cancer pain [1]. Bone metastases from elsewhere are the most common form of bone cancer pain, where the relative incidence of bone metastases is 60–70% in advanced breast cancer [2,3,4]. BCP is, to some extent, a unique type of chronic pain that is characterized by nociceptive and neuropathic components and is manifested clinically as a mixture of steady ongoing pain and breakthrough pain caused by weight-bearing or movement [2,5]. Unfortunately, current therapeutic options for BCP are quite limited and nonspecific with considerable side effects [6]; thus, novel strategies based on its pathogenesis are needed to effectively improve these patients’ living quality.

MicroRNAs (miRNAs) are small non-coding RNAs with a length of about 20 to 25 nucleotides that can bind to specific sequences in the 3′ untranslated region (UTR) of the target mRNAs (seed region) and cause translational repression or mRNA degradation. The role of miRNA in chronic pain mechanisms in various animal models has been researched in recent decades [7], and some miRNA targets may be involved in BCP development, such as miR-124 [8] and miR-135-5p [9]; however, further studies are still necessary to explore the role of other miRNAs in BCP pathogenesis. In the present study, miR-155-5p was significantly upregulated in the lumbar enlargement of the spinal cord of BCP rats, as found using RNA-seq analysis. Possibly by activating microglia [10,11], miR-155-5p is involved in the spinal mechanism of neuropathic pain [12,13], and the suppression of miR-155-5p could attenuate the neuropathic pain caused by diabetes mellitus [14] and spinal cord injury [15]. However, the role of miR-155-5p in BCP has rarely been investigated.

It is well acknowledged that neuroinflammation in the central nervous system (CNS), which is characterized by the activation of glial cells, such as microglia and astrocytes, plays an important role in the regulation of chronic pain [16,17,18]. Microglia, which are the tissue-resident macrophages of the CNS, change into a reactive hypertrophic shape from a normal ramified surveillance shape and proliferate markedly (referred to as microgliosis), and produce a variety of pro-inflammatory factors, such as TNF-α (tumor necrosis factor), IL-1β (interleukin 1 beta), and IL-6 (interleukin 6), during chronic pain [19,20], causing aberrant hyperexcitability of neurons [20] and regulating synaptic transmission in the spinal cord dorsal horn [19]. Furthermore, the activated microglia in the spinal cord dorsal horn could also degrade the extracellular perineuronal nets, thereby enhancing the activity of projection neurons and inducing pain hypersensitivity [21]. When it comes to BCP, some researchers found that spinal microglial activation contributes to BCP development and inhibiting microglia activation using pharmacological methods attenuates BCP [22,23,24]; however, others demonstrated that microglial activation is not necessary for BCP pathogenesis [25,26]. Thus, in this study, we attempted to verify the role of microglial activation in the spinal cord of BCP rats.

Taken together, in the current study, we tried to test the hypothesis that the aberrant expression of miR-155-5p in the spinal cord, which was filtered out using RNA-seq (RNA sequencing), mediates microglial activation, possibly by inhibiting *Sgk3*, and finally promotes BCP development.

## 2. Materials and Methods

### 2.1. Bone Cancer Pain Model

Female Sprague Dawley (SD) rats (weights 80–100 g and 200–250 g) were obtained from Hunan SJA Laboratory Animal Co., Ltd. (Changsha, China)., and were housed in a thermostatic room maintained at 22 ± 2 °C with a 12 h dark/light cycle and had free access to food and water. The BCP model was constructed as previously described [27]. Female SD rats (80–100 g) received an intraperitoneal inoculation of Walker 256 breast carcinoma cells. After 7–10 days, Walker 256 cells for inoculation were extracted from the ascitic fluid and resuspended to a final concentration of 1 × 10^7^ cells/mL. Then, female SD rats (200–250 g) were anesthetized with isoflurane. The left hindlimb of each rat was shaved and disinfected with a povidone–iodine solution. A minimal incision was made across the femoral–tibial region and a 22-gauge needle was inserted into the tibial intramedullary canal. A volume of 10 μL heat-killed cells (sham group) or the prepared Walker 256 cells (1 × 10^5^ cells) (BCP group) were slowly injected into the bone cavity. The cavity was immediately sealed using bone wax after the syringe was removed, and the incision was sutured with a 3-0 silk thread and disinfected.

All experiments were approved by the Animal Care and Use Committee of Central South University (no. 2020sydw0168) and performed following the International Association for the Study of Pain guidelines.

### 2.2. Behavioral Assessment

As described earlier [28], the paw withdrawal mechanical threshold (PWMT) of the left hind paw was measured to evaluate mechanical hyperalgesia. Briefly, rats were individually placed in plastic boxes (22 × 12 × 12 cm^3^) with a metal mesh floor and acclimated for 30 min. Von Frey monofilaments (Aesthesio, San Jose, CA, USA), starting with 0.4 g and ending with 15 g in ascending order, were applied to the plantar surface of the left hind paw. Quick paw withdrawal or licking was considered a positive response. Gradually increased stimulation was applied until three positive responses were evoked out of five applications, and the lowest Von Frey force was recorded as the PWMT. Measurements were repeated three times and averaged in every rat at intervals of 10 min.

### 2.3. Tissue Preparation

After the behavioral assessments were completed, the rats were euthanized. The left tibia was preserved in 4% paraformaldehyde, decalcified in 10% EDTA, and sectioned and stained with hematoxylin and eosin (H&E). The lumbar spinal cord (L4–L6) was quickly harvested on ice and snap-frozen in liquid nitrogen for miRNA sequencing, RT-qPCR, and Western blot detection. The rest of the rats in each group were transcardially perfused with 0.1 M PBS and 4% paraformaldehyde (pH 7.4) for prefixation. The lumbar spinal cords were taken out and immersed in 4% polyformaldehyde for post-fixation and frozen for fluorescence in situ hybridization (FISH) and immunofluorescence.

### 2.4. Sequencing and Analysis of miRNAs

Total RNA was extracted from the lumbar spinal cord tissue using an RNeasy Mini Kit (Cat# 74106, Qiagen, Hilden, Germany) according to the manufacturer’s instructions, followed by RNA purification, library construction using NEBNext^®^ Multiplex Small RNA Library Prep Set for Illumina (Cat# E7580S, NEB, Ipswich, MA, USA), and miRNA sequencing that was performed on Illumina NovaSeq 6000 system conducted by Shanghai Biotechnology Corporation, generating more than 30 million 50-base single-end reads per sample. Then, raw reads were processed using the FASTX Tool kit (version 0.0.13, http://hannonlab.cshl.edu/fastx_toolkit, accessed on 15 February 2021) to filter out the low-quality reads, as well as sequences with 3′ primers, with 5′ primers, with poly-A tails, or with insert tags to obtain clean reads. By confining the length to 18–40 nucleotides, clean reads were mapped to a rat reference genome (Rnor 6.0) using Bowtie (v2.2.3, http://bowtie-bio.sourceforge.net, accessed on 15 February 2021) to obtain different types of small RNA, among which known miRNAs were identified via their lengths (21–22 nucleotides) and the annotation in miRbase (version: 22.0) and quantified using TPM (transcripts per kilobase of exon model per million mapped reads); then, edgeR was used to identify differentially expressed miRNAs.

### 2.5. N9 Cell Culture and Treatment

The N9 cell line was a gift from the Chinese Academy of Sciences. The cells were cultured in Dulbecco’s modified Eagle’s medium (DMEM) containing 10% fetal bovine serum (Gibco, New York, NY, USA) and 1% penicillin/streptomycin. Cells were maintained at 37 °C in a humidified chamber containing 5% CO_2_ and 95% air. The medium was refreshed every two days. Cells were divided and seeded in 6-well cell culture plates. The cells in the LPS group were treated with lipopolysaccharide (LPS, *E. coli* O111, Sigma, St. Louis, MO, USA), with a final concentration of 1000 ng/mL. The control (Con) group was given the same volume of sterile PBS. Cells were harvested after 24 h of LPS treatment.

### 2.6. Real-Time Quantitative PCR (RT-qPCR)

Total RNA was extracted using TRIzol (Invitrogen, Waltham, MA, USA), and cDNA was generated using the All-in-One^TM^ miRNA First-Strand cDNA Synthesis Kit (GeneCopoeia, Guangzhou, China) for the miRNA analysis and the All-in-One^TM^ mRNA First-Strand cDNA Synthesis Kit (GeneCopoeia, China) for the mRNA analysis according to the manufacturer’s instructions. The All-in-One^TM^ miRNA qPCR Kit or the All-in-One^TM^ mRNA qPCR Mix (GeneCopoeia, China) was used for the qPCR. For the miRNA qPCR, 3.9 μL double-distilled water, 10 μL 2 × All-in-One qPCR Mix, 2 μL All-in-One^TM^ miRNA qPCR Primer (2 μM), 2 μL Universal Adaptor PCR Primer (2 μM), 0.1 μL ROX Reference Dye (30 μM), and 2 μL of complementary DNA (cDNA) were successively added to each well of a 96-well real-time polymerase chain reaction plate (Axygen, Union City, CA, USA). For the mRNA qPCR, 5.6 μL double-distilled water, 10 μL 2 × All-in-One^TM^ qPCR Mix, 2 μL qPCR Primer (2 μM), 0.4 μL ROX Reference Dye (30 μM), and 2 μL of complementary DNA (cDNA) were successively added to each well of a 96-well real-time polymerase chain reaction plate (Axygen, Union City, CA, USA). Each sample was tested in duplicate. Then, qPCR assays were carried out using the Applied Biosystems 7300 fast thermocycler. For the miRNA qPCR, the PCR reactions were incubated at 95 °C for 10 min; followed by 40 cycles of 95 °C for 10 s, 60 °C for 20 s, and 72 °C for 10 s; and then by melting curve analysis. For the mRNA qPCR, the PCR reactions were incubated at 95 °C for 10 min; followed by 40 cycles of 95 °C for 10 s, 60 °C for 20 s, and 72 °C for 15 s; and then by melting curve analysis. U6 or GAPDH was used as the internal reference. Some primers were ordered from GeneCopoeia, and the catalog numbers were as follows: U6 primer—cat# RmiRQP9003, rno-miR-155-5p primer—cat# RmiRQP0890, rat *Sgk3* primer—cat#CS-RQP0002, U6 primer—mouse#MmiRQP9002, mmu-mir-155-5p primer—mouse#MmiRQP0890, and mouse *Sgk3* primer—mouse#MQP025071; others were from Sangon Biotech Co., Ltd. (Shanghai, China), and the sequences are shown in Supplementary Table S1.

### 2.7. Western Blot Analysis

Spinal cord samples were lysed using ice-cold RIPA buffer with protease inhibitors (Beyotime, China), followed by centrifugation at 12,000 g/min for 30 min at 4 °C. Supernatants were collected, and protein concentrations were determined with the BCA protein quantitative analysis kit (Beyotime, Shanghai, China). After the addition of a loading buffer and thermal denaturation at 99 °C for 5 min, proteins were separated using 8% sodium dodecyl sulfate–polyacrylamide (SDS-PAGE) gels and transferred to PVDF membranes (Millipore, Bedford, MA, USA) at 120 mA for 60 min. Membranes were blocked with 5% nonfat milk for 2 h at room temperature and were incubated with a rabbit anti-SGK3 antibody (1:1000, Servicebio, Wuhan, China) and rabbit anti-GAPDH antibody (1:1000, Proteintech, Rosemont, IL, USA) at 4 °C overnight. Following three washes with TBST for 10 min, membranes were incubated with the horseradish-peroxidase-conjugated secondary antibodies (1:5000, Absin, Shanghai, China) for 1 h at room temperature. Immunoreactive proteins were visualized using super ECL Western blot detection reagents (Millipore, Burlington, MA, USA). The intensity of each band was quantified using ImageJ software (National Institutes of Health, Bethesda, MD, USA) and normalized to that of GAPDH.

### 2.8. Fluorescence In Situ Hybridization (FISH) and Immunofluorescence Assay

Frozen sections were prepared according to the FISH kit protocol (Boster, Wuhan, China). Oligonucleotide probes complementary to rno-miR-155-5p were purchased from Servicebio (Wuhan, China). The probe was labeled with digoxin, and the sequence was as follows: 5′-DIG-ACCCCTATCACGATTAGCATTAA-DIG-3′; scrambled locked nucleic acid (LNA) probes were used as a negative control. Sections were fixed in 4% paraformaldehyde for 10 min and digested with proteinase K (20 mg/mL) (Boster, Pleasanton, CA, USA) for 15 min at 37 °C. Then, prehybridization was conducted with prehybridization buffer (Boster, USA) for 1 h at 37 °C. The miR-155-5p probe was diluted with hybridization buffer to 1 mmol/L and incubated for 16 h at 37 °C. After hybridization, the excess probe was washed in 2 × SSC for 10 min at 37 °C, 1 × SSC two times for 5 min each at 37 °C, and 0.5 × SSC for 10 min at room temperature, and sections were blocked at room temperature for 30 min. Sections were then incubated with DyLight-488-conjugated IgG fraction monoclonal mouse anti-digoxin for 50 min at 37 °C. To identify the protein co-expressed with miR-155-5p, the sections used for FISH were further incubated overnight with anti-Iba1 (1:500, Servicebio, Wuhan, China) for 16 h at 4 °C. Finally, the sections were rinsed in 0.01 M PBS 3 times and then incubated with Alexa-Fluor-594-conjugated goat anti-rabbit antibody (1:200, Jackson, West Grove, PA, USA).

For immunofluorescence, frozen sections were blocked with a blocking buffer containing 5% normal goat serum and 0.3% Triton X-100 for 1 h at room temperature and were then incubated with mouse anti-Iba1 (1:200 dilution; Servicebio, Wuhan, China) and rabbit anti-SGK3 (1:100 dilution; Bioss, Shanghai, China) antibodies overnight at 4 °C. After incubation with Alexa-Fluor-488-conjugated goat anti-mouse IgG (1:200 dilution; Servicebio, Wuhan, China) and Cy3-conjugated goat anti-rabbit IgG (1:200 dilution; Servicebio, Wuhan, China) antibodies, the sections were sealed with a DAPI-containing antifade agent.

Images were captured using a microscope equipped with a computer (Nikon, Tokyo, Japan).

### 2.9. Dual-Luciferase Reporter Assay

The partial wild-type 3′ UTR of *Sgk3* (NM_001376043.1) was amplified using PCR and subsequently mutated using site-directed mutagenesis. After being sequenced, the correct recombinant vector was double-digested with restriction enzymes *Mlu I* and *Hind III* to release the sequence of interest, which was inserted into the empty luciferase reporting vector (pMIR-REPORTTM Luciferase, 6470bp, Ambion Life Technologies, Carlsbad, CA, USA). HEK-293T cells were cultured in Dulbecco’s modified Eagle’s medium (C11965500BT, Gibco) containing 10% fetal bovine serum (FBS) (10270-106, Gibco) for 24 h. Then, 0.2 µg *Sgk3* WT or MUT recombinant plasmid (Firefly luciferase vector) and 0.01 µg pRL-CMV (Ranilla luciferase vector) were co-transfected with 25 μL of either miR-155-5p mimics or nontargeting control mimics (100 nM) using liposome (Lipofectamine^®^ 2000, Invitrogen Life Technologies). Forty-eight hours later, Firefly and Renilla luciferase activities were quantified using the Dual-Luciferase Reporter Assay System (E1960, Promega, Madison, WI, USA) and normalized to the control group.

### 2.10. Lentivirus Infection In Vitro and In Vivo

To inhibit miR-155-5p, a recombinant lentivirus-mediated miR-155-5p sponge was designed and constructed in which a partially modified complementary strand of miR-155-5p was inserted six times into vector GV309 (vector size: 11.4 kb) with a frame structure of hU6-MCS-Ubiquitin-EGFP-IRES-puromycin. A scrambled sequence was inserted into the negative control lentivirus. The miR-155-5p sponge lentivirus and negative control lentivirus were identically packaged in 293T cells with a final titer of approximately 6 × 10^8^ TU (transducing units)/mL. The lentivirus vector synthesis, identification, sequencing, extraction, and packaging were conducted by Shanghai Genechem Corporation.

The N9 cells in 6-well plates were divided into three groups: the control (Con + NC) group, the LPS + sponge-NC (LPS + NC) group, and the LPS + miR-155-5p sponge (LPS + sponge) group. After the same volume of lipopolysaccharide (final concentration of 1000 ng/mL) (LPS + NC group and LPS + sponge group) or PBS (Con + NC group) treatment for 24 h, the N9 cells were transfected with the miR-155-5p sponge or sponge-NC for 24 h. Then, the cells were collected for subsequent experiments.

The rats were randomly divided into the following 3 groups: BCP + miR-155-5p sponge (BCP + sponge), BCP + sponge-NC (BCP + NC), and sham + sponge-NC as a control (sham + NC). On the seventh day of bone cancer pain model establishment, after being disinfected with iodophor, a small incision was made in the skin of the back of the rats under isoflurane anesthesia. Then, a PE-10 catheter (ID = 0.28 mm, OD = 0.61 mm, Smiths Medical, Plymouth, MN, USA) was cannulated through the L5/L6 intervertebral space to the subarachnoid space based on a previously described method [29]. For each intrathecal administration, 10 μL of miR-155-5p sponge or sponge-NC was injected into the intrathecal catheter using a microinjection syringe.

### 2.11. Statistical Analysis

Statistical analysis and graphing were performed using GraphPad Prism (version 8.0; GraphPad Software, Inc., San Diego, CA, USA). All quantitative data are expressed as the mean ± standard deviation (SD). Differences between two groups were analyzed using the unpaired Student’s *t*-test. Differences between more than two groups were analyzed using one-way ANOVA followed by Dunnett’s multiple comparisons test. The behavioral test data were compared using two-way ANOVA for repeated measurement data, followed by Tukey’s post hoc test. A *p*-value < 0.05 was considered statistically significant.

## 3. Results

### 3.1. Mechanical Hyperalgesia Developed in BCP Rats and Differentially Expressed miRNAs Were Filtered Out Using RNA-seq

**Figure 1:**
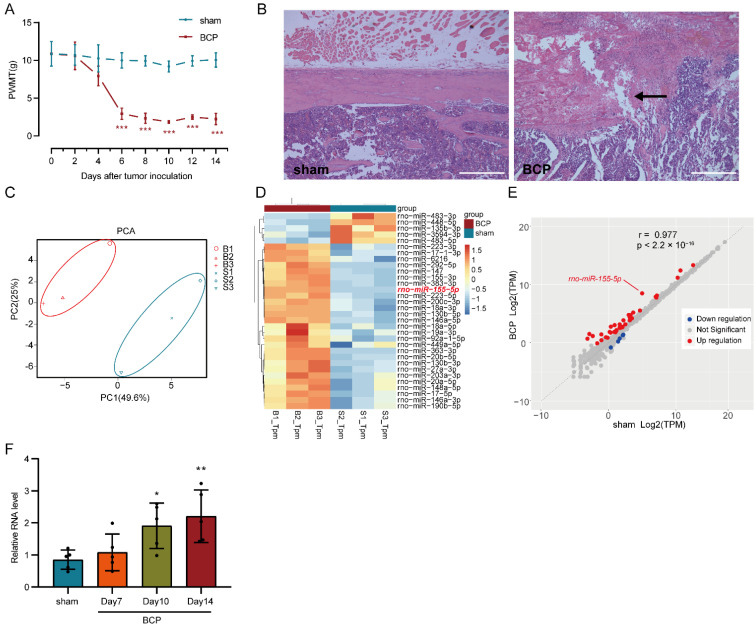
Establishment of a bone cancer pain (BCP) model and transcriptome sequencing (RNA-seq) of miRNA. (**A**) Mechanical hyperalgesia was induced in bone cancer pain rats six days after Walker 256 carcinoma cell inoculation and was maintained to the end of the experiment. *n* = 5, *** *p* < 0.001 vs. the sham-operated group. (**B**) Bone destruction was found in the BCP rats using H&E staining of tibia sections, the black arrow indicates the discontinuity of cortical bone (scale bar: 500 μm). (**C**) Sham-operated and bone cancer pain samples (marked by different colors) were separated in the principal component analysis (PCA) of the general gene expression value. B1, B2, and B3 represent biological replicates of the BCP group, while S1, S2, and S3 represent biological replicates of the sham-operated group. (**D**) After neglecting unannotated and extremely lowly expressed miRNAs, 31 differentially expressed miRNAs (26 upregulated miRNAs and 5 downregulated miRNAs), which were filtered using the threshold of |log2FC|> 0.8 and an adjusted *p* < 0.05, were listed and clustered in a heatmap. (**E**) Differentially expressed miRNAs (marked using different colors) are shown in the scatter plot. (**F**) RT-qPCR of miR-155-5p verified its gradually increased expression in the BCP rats. *n* = 5, * *p* < 0.05, ** *p* < 0.01 vs. the sham-operated group.

In this study, the bone cancer pain model was established via intramedullary injection of Walker 256 carcinoma cells into the left tibia of rats; then, the paw withdrawal mechanical threshold (PWMT) of the left hind limb was measured to evaluate the mechanical hyperalgesia. There was no significant difference in the PWMT baseline between the sham-operated and BCP rats; then, the PWMT of BCP rats decreased persistently from the 6th day after the tumor cell injection and was maintained at a relatively low level, while the PWMT of the sham-operated rats remained unchanged. The differences in PWMT between the two groups were significant on days 6, 8, 10, 12, and 14 after the tumor cells injection (multiple comparisons: on days 6, 8, 10, 12, and 14; all *p* < 0.0001; BCP vs. sham-operated group; Figure 1A), indicating that the BCP rats displayed mechanical hyperalgesia. Pathologic examination of the left tibia revealed that severe bone destruction was caused by tumor inoculation in the BCP rats compared with the sham-operated rats (Figure 1B).

miRNA-seq of the lumbar spinal cord 14 days after tumor cells injection was employed to detect differentially expressed miRNAs in bone cancer pain and raw data were deposited in the Gene Expression Omnibus (GEO) database of the NCBI (National Center for Biotechnology Information) with an accession number is GSE200307. Among all samples, the ratio of clean reads to raw reads was more than 98.0% (Table S2); in the clean reads with 18–40 nucleotides, the ratio of miRNA was more than 86.1% (Table S3). Principal component analysis (PCA) of the general expression of all miRNAs in six samples showed that the BCP group was separated from the sham-operated group (Figure 1C). Among the 753 known miRNAs and 114 novel miRNAs detected, 31 differentially expressed miRNAs (26 upregulated miRNAs and 5 downregulated miRNAs) were identified, clustered in a heatmap (Figure 1D), and displayed in a scatter plot (Figure 1E), which were filtered using the threshold of |log2FC| > 0.8, an adjusted *p* < 0.05, and neglecting unannotated and extremely lowly expressed miRNAs. Despite the relatively loose criteria being adopted, not so many differentially expressed miRNAs were detected, which was possibly due to the low expression level of miRNA, incomplete annotation in the rat miRNA database, and the detection sensitivity of the current technology. miR-155-5p is one of the upregulated miRNAs (Figure 1D,E) and was subsequently verified in another patch of rats using a traditional RT-qPCR assay. The expression level of miR-155-5p was gradually increased and the differences were statistically significant 10 days and 14 days after the tumor inoculation (multiple comparisons: adjusted *p* = 0.0444, 10 days of BCP vs. sham-operated group; adjusted *p* = 0.0098, 14 days of BCP vs. sham-operated group; Figure 1F), indicating its possible roles in bone cancer pain pathogenesis.

### 3.2. miR-155-5p Could Be Expressed in the Cytoplasm of Microglia in the Lumbar Spinal Cord of BCP Rats

**Figure 2:**
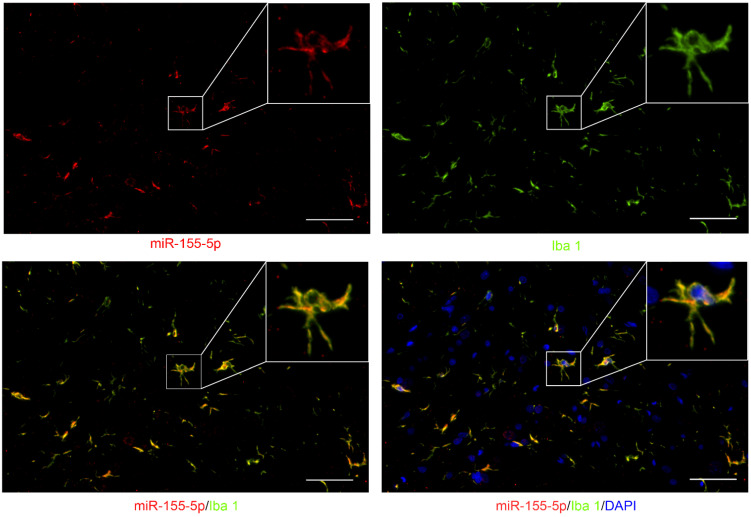
Fluorescence in situ hybridization (FISH) of miR-155-5p (green) showed its co-localization with Iba1 (red, a marker of microglia) and outside of the nucleus (marked with DAPI, blue) (scale bar: 10 μm) in the lumbar spinal cord of BCP rats.

To elucidate the expression pattern of miR-155-5p in the spinal cord, fluorescence in situ hybridization (FISH) of miR-155-5p was performed and co-localization with Iba1 indicated its expression in microglia (bottom left of Figure 2), while no co-localization with DAPI indicated its expression in the cytoplasm (bottom right of Figure 2).

### 3.3. Microglia Were Dramatically Activated and Accompanied by the Increase in Inflammatory Cytokines in BCP Rats

**Figure 3:**
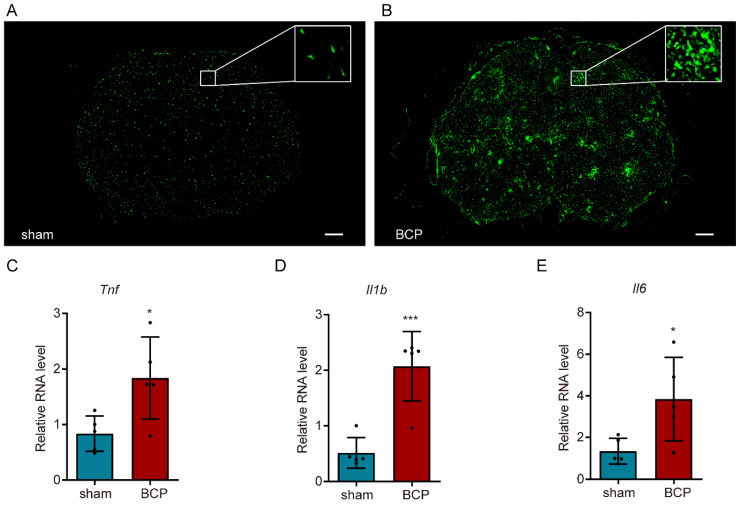
Activation of microglia in the lumbar spinal cord of bone cancer pain rats. (**A**,**B**) Immunofluorescence of Iba1 showed the morphology and number of activated microglia (scale bar: 200 μm). (**C**–**E**) RT-qPCR analysis of inflammatory factors showed the surge of *Tnf*, *Il1b*, and *Il6* in bone cancer pain. *n* = 5, * *p* < 0.05, *** *p* < 0.001 vs. the sham-operated group.

To clarify the cellular state of microglia in bone cancer pain, immunofluorescence of Iba1 was conducted in the lumbar spinal cord in the sham-operated and BCP rats. Apparent microgliosis—including a proliferation in cell number, a profound morphologic change from a normal ramified surveillance shape to a reactive hypertrophic ameboid shape with enlarged cell bodies, and shortened processes—were shown in the BCP rats (Figure 3A,B). The large increase in inflammatory cytokines production could be viewed as an indicator of microglia activation. Thus, RT-qPCR was performed to detect the expression of inflammatory factors and the results showed that the expressions of *Tnf*, *Il1b*, and *Il6* in the BCP group were significantly higher than those in the sham-operated group (unpaired *t*-test of *Tnf*: *p* = 0.0238, BCP vs. sham-operated group; unpaired *t*-test of *Il1b*: *p* = 0.0009, BCP vs. sham-operated group; unpaired *t*-test of *Il6*: *p* = 0.0287, BCP vs. sham-operated group; Figure 3C–E), indicating the involvement of neuroinflammation in the development of bone cancer pain.

### 3.4. The Expressions of miR-155-5p, Tnf, Il1b, and Il6 Were Increased in N9 Cells Treated with LPS

To imitate the inflammatory activated state of microglia that was observed in bone cancer pain, N9 cells were treated with LPS at a concentration of 1000 ng/mL. RT-qPCR analyses of miR-155-5p, *Tnf*, *Il1b*, and *Il6* expression showed that they were upregulated in the LPS group (unpaired *t*-test of miR-155-5p: *p* = 0.0093, LPS vs. Con; unpaired *t*-test of *Tnf*: *p* < 0.0001, LPS vs. Con; unpaired *t*-test of *Il1b*: *p* < 0.0001, LPS vs. Con; unpaired *t*-test of *Il6*: *p* < 0.0001, LPS vs. Con; Supplementary Figure S1), which were all consistent with the results of the animal experiments in vivo.

### 3.5. Knockdown of miR-155-5p Alleviated the Upregulation of Tnf, Il1b, and Il6 in LPS-Treated N9 Cells In Vitro

**Figure 4:**
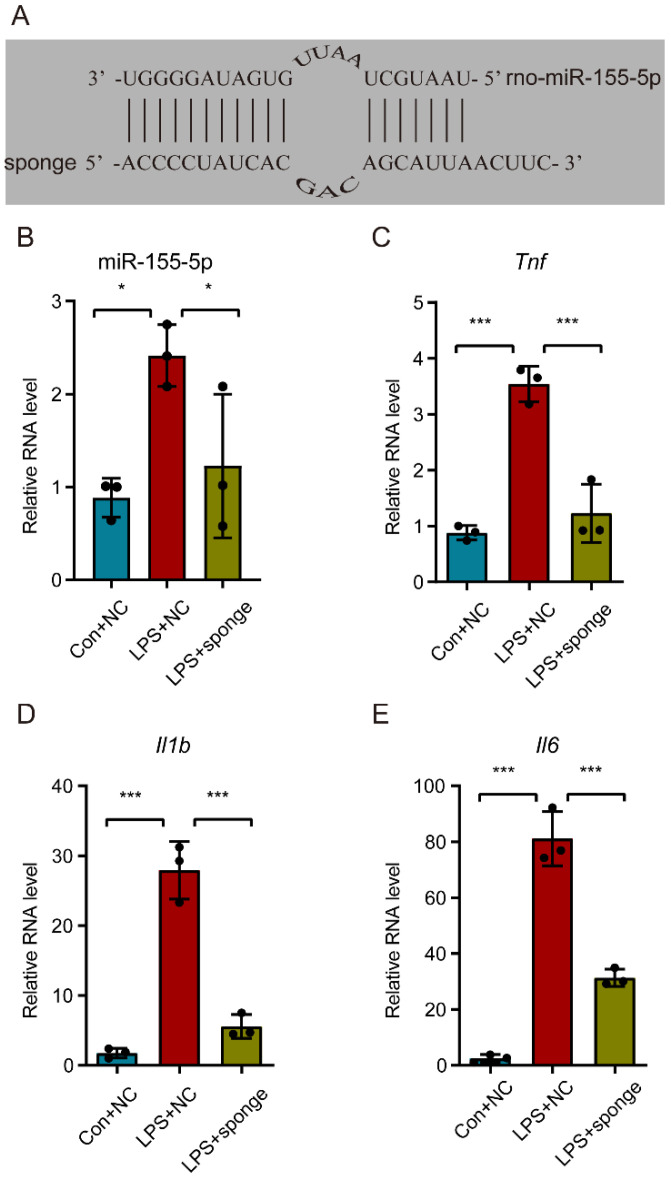
Knockdown of miR-155-5p alleviated the inflammatory storm in microglia caused by the LPS treatment. (**A**) The core sequence of the recombinant lentivirus-mediated miR-155-5p sponge was designed to be partially complementary to that of miR-155-5p. (**B**) The lentivirus-mediated miR-155-5p sponge knocked down the expression of miR-155-5p in LPS-treated microglia. *n* = 3, * *p* < 0.05, LPS + NC vs. Con + NC; * *p* < 0.05, LPS + sponge vs. LPS + NC. (**C**–**E**) The miR-155-5p sponge alleviated the surge of *Tnf*, *Il1b*, and *Il6* induced by LPS. *n* = 3, *** *p* < 0.001, LPS + NC vs. Con + NC; *** *p* < 0.001, LPS + sponge vs. LPS + NC.

In order to explore the role of miR-155-5p in the inflammatory active state of microglia, a recombinant lentivirus-mediated miR-155-5p sponge was designed with a partially modified complementary strand to that of miR-155-5p (Figure 4A). RT-qPCR assay results confirmed that the miR-155-5p sponge could effectively knock down miR-155-5p in LPS-treated N9 cells (multiple comparisons: adjusted *p* = 0.0170, LPS + NC vs. Con + NC; adjusted *p* = 0.0475, LPS + NC vs. LPS + sponge; Figure 4B). Subsequently, detection of the expression of inflammatory factors showed that the upregulation of *Tnf*, *Il1b*, and *Il6* were alleviated in LPS-treated N9 cells (multiple comparisons of *Tnf*: adjusted *p* = 0.0002, LPS + NC vs. Con + NC; adjusted *p* = 0.0004, LPS + NC vs. LPS + sponge; multiple comparisons of *Il1b*: adjusted *p* < 0.0001, LPS + NC vs. Con + NC; adjusted *p* < 0.0001, LPS + NC vs. LPS + sponge; multiple comparisons of *Il6*: adjusted *p* < 0.0001, LPS + NC vs. Con + NC; adjusted *p* < 0.0001, LPS + NC vs. LPS + sponge; Figure 4C–E), indicating that miR-155-5p may play a regulatory role in a microglial-activation-mediated inflammatory storm.

### 3.6. Sgk3 May Have Been a Direct Target of miR-155-5p In Vitro

**Figure 5:**
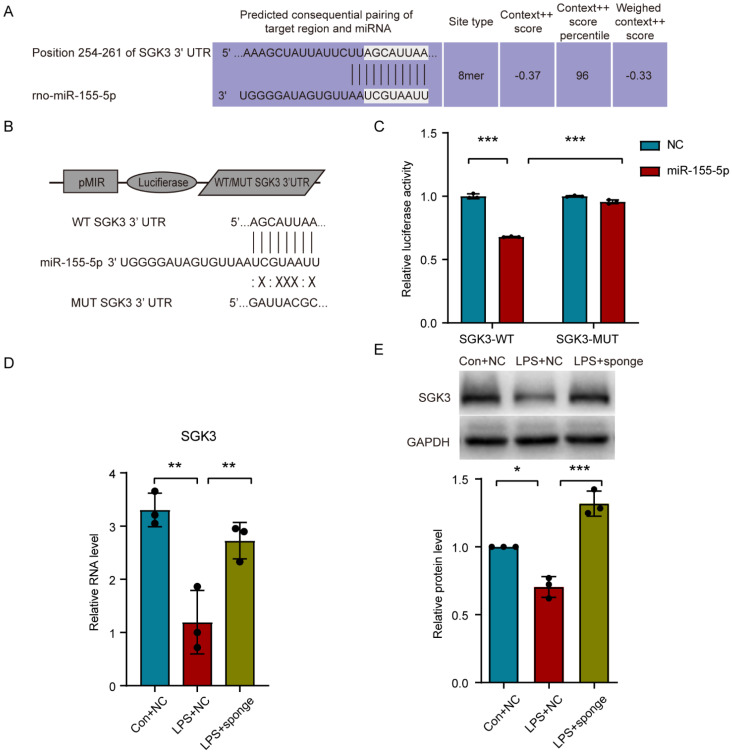
miR-155-5p suppressed the *Sgk3* expression in vitro. (**A**) The probable pairing sequences of miR-155-5p and the 3′ untranslated region (UTR) of *Sgk3* (seed region shown in grey boxes). (**B**) The structure of pMIR-REPORTTM Luciferase system and sequences of wild type and mutated 3′ UTR of *Sgk3*. (**C**) Dual-luciferase activity reporter assay showed that miR-155-5p mimics decreased the luciferase activity in 293T cells transfected with *Sgk3*-WT, but not in the MUT vector. *n* = 3, *** *p* < 0.001, *Sgk3*-WT + miR-155-5p vs. *Sgk3*-WT + NC; *** *p* < 0.001, *Sgk3*-WT + miR-155-5p vs. *Sgk3*-MUT + miR-155-5p. (**D**,**E**) Knockdown of miR-155-5p using a miR-155-5p sponge restored the expression of *Sgk3* suppressed by LPS treatment assayed using RT-qPCR (**D**) and Western blot (**E**). *n* = 3, * *p* < 0.05, ** *p* < 0.01, LPS + NC vs. Con + NC; ** *p* < 0.01, *** *p* < 0.001, LPS + sponge vs. LPS + NC.

By referring to Targetscan [30] (Release 7.2, http://www.targetscan.org/vert_72/, accessed on 15 February 2021), serum/glucocorticoid regulated kinase family, member 3 (*Sgk3*), which is a member of the Ser/Thr protein kinase family, was speculated as a direct target of miR-155-5p with nearly complete pairing sequences in the 3′ untranslated region (UTR) and that of miR-155-5p (Figure 5A). Additionally, the immunofluorescence stain of SGK3 in the spinal cord showed that it could also be expressed in spinal microglia (Supplementary Figure S2), similarly to miR-155-5p. To verify their possible interaction, we designed a dual-luciferase activity reporter assay in which the wild-type and mutated 3′ UTR of *Sgk3* was inserted into pMIR-REPORTTM Luciferase (Figure 5B). After being co-transfected with miR-155-5p mimics or negative control mimics, the relative luciferase activity significantly decreased in the miR-155-5p group compared with that in the control group (*Sgk3*-WT + NC) (multiple comparisons: adjusted *p* < 0.0001, WT + NC vs. WT + miR-155-5; Figure 5C); mutation of the predicted sites in the 3′-UTR of *Sgk3* (MUT) significantly reduced the suppression of the luciferase signal induced by miR-155-5p mimics (multiple comparisons: adjusted *p* < 0.0001, WT + miR-155-5p vs. MUT + miR-155-5p; Figure 5C). These results indicated that miR-155-5p could regulate the expression of *Sgk3* through complementary binding to this site, which is consistent with previous research [31]. To further confirm the regulatory role of miR-155-5p regarding *Sgk3*, the expression of *Sgk3* was assayed using RT-qPCR and Western blot, which both showed that the knockdown of miR-155-5p using a miR-155-5p sponge could restore the expression of *Sgk3* suppressed by the LPS treatment (multiple comparisons of mRNA level: adjusted *p* = 0.0019, LPS + NC vs. Con + NC; adjusted *p* = 0.0091, LPS + NC vs. LPS + sponge; Figure 5D) (multiple comparisons of protein level: adjusted *p* = 0.0036, LPS + NC vs. Con + NC; adjusted *p* < 0.0001, LPS + NC vs. LPS + sponge; Figure 5E), indicating miR-155-5p may have had an inhibiting effect on *Sgk3*.

### 3.7. Inhibiting miR-155-5p Altered the Expression of Sgk3, Alleviated Spinal Cord Inflammation, and Attenuated Mechanical Hyperalgesia in BCP Rats

**Figure 6:**
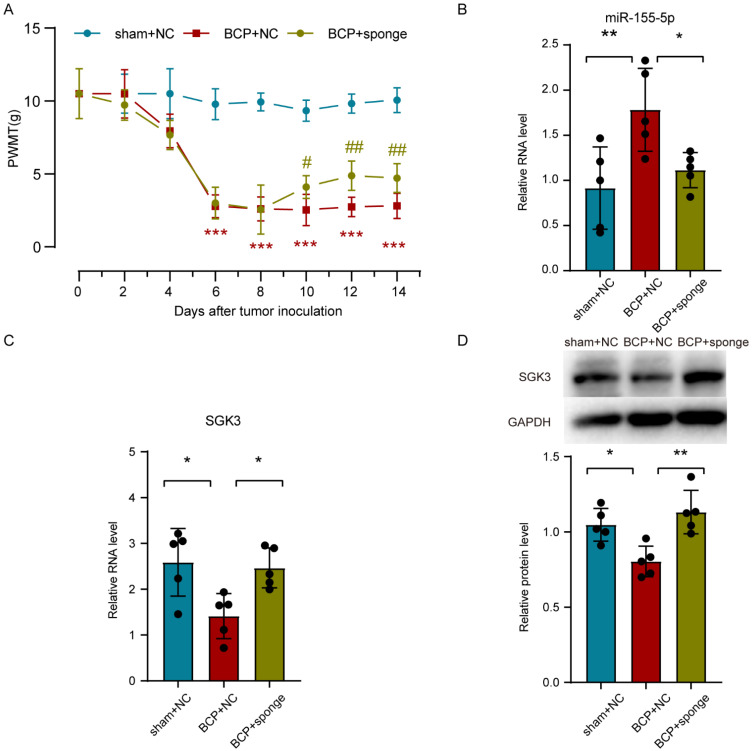
Intrathecal administration of a miR-155-5p sponge knocked down miR-155-5p expression, restored *Sgk3* expression, and attenuated mechanical hyperalgesia in the bone cancer pain rats. (**A**) The mechanical hyperalgesia was attenuated in the bone cancer pain rats after the miR-155-5p sponge administration. *n* = 5, *** *p* < 0.001 vs. the sham + NC group; *n* = 5, # *p* < 0.05, ## *p* < 0.01 vs. the BCP + NC group. (**B**) The lentivirus-mediated miR-155-5p sponge knocked down the expression of miR-155-5p in vivo. *n* = 5, ** *p* < 0.01 vs. the sham + NC group; * *p* < 0.05 vs. the BCP + NC group. (**C**,**D**) Administration of the miR-155-5p sponge restored the expression of *Sgk3* suppressed in bone cancer pain according to an assay using RT-qPCR (**C**) and Western blot (**D**). *n* = 5, * *p* < 0.05 vs. the sham + NC group; * *p* < 0.05, ** *p* < 0.01 vs. the BCP + NC group.

To clarify the role of miR-155-5p in bone cancer pain, the miR-155-5p sponge was administrated intrathecally in the bone cancer pain rats. A behavioral test showed that the PWMT of the BCP rats was partially recovered by inhibiting miR-155-5p (multiple comparisons: on days 6, 8, 10, 12, and 14; all adjusted *p* < 0.0001; BCP + NC vs. sham + NC) (on day 10, adjusted *p* = 0.030, BCP + NC vs. BCP + sponge) (on day 12, adjusted *p* = 0.0037, BCP + NC vs. BCP + sponge) (on day 14, adjusted *p* = 0.0100, BCP + NC vs. BCP + sponge) (Figure 6A). Then, an assay of miR-155-5p expression showed that the miR-155-5p sponge could effectively knock down miR-155-5p in the lumbar spinal cord of bone cancer pain rats (multiple comparisons: adjusted *p* = 0.0079, BCP + NC vs. sham + NC; adjusted *p* = 0.0346, BCP + NC vs. BCP + sponge; Figure 6B). Consistent with the in vitro study, inhibiting miR-155-5p restored the expression of *Sgk3* suppressed in the bone cancer pain rats assayed using RT-qPCR (multiple comparisons: adjusted *p* = 0.0128, BCP + NC vs. sham + NC; adjusted *p* = 0.0238, BCP + NC vs. BCP + sponge; Figure 6C) and Western blot (multiple comparisons: adjusted *p* = 0.0137, BCP + NC vs. sham + NC; adjusted *p* = 0.0018, BCP + NC vs. BCP + sponge; Figure 6D), indicating the regulatory effect of miR-155-5p.

**Figure 7:**
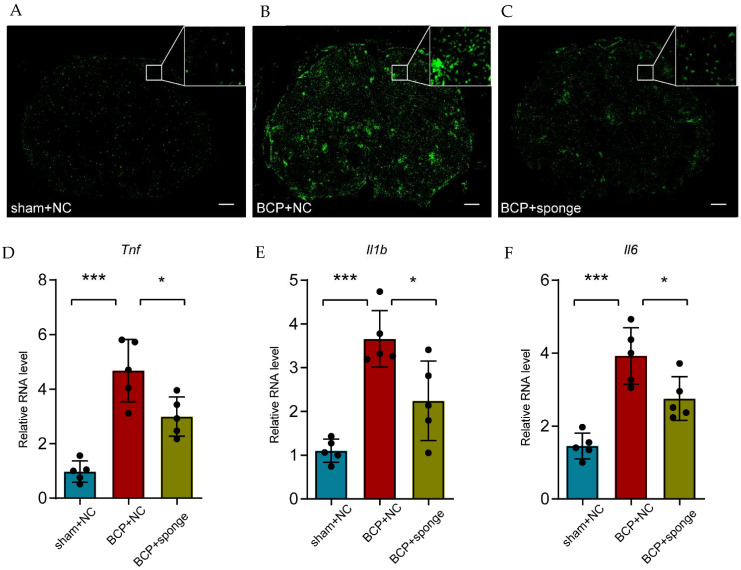
Intrathecal administration of a miR-155-5p sponge alleviated microglial activation in the bone cancer pain rats. (**A**–**C**) Immunofluorescence of Iba1 showed the morphology and number of activated microglia in different treatments (scale bar: 200 μm). (**D**–**F**) The administration of a miR-155-5p sponge alleviated the inflammatory factors (i.e., *Tnf*, *Il1b*, and *Il6*) that were increased in the bone cancer pain condition. *n* = 5, *** *p* < 0.001 vs. the sham + NC group; * *p* < 0.05 vs. the BCP + NC group.

Meanwhile, the activation state of microglia was visualized using immunofluorescence in which the miR-155-5p sponge administration notably relieved the microgliosis (Figure 7A–C). Consistent with in vitro study, the detection of inflammatory factors showed that the expression of *Tnf*, *Il1b*, and *Il6* was partially alleviated in the lumbar spinal cord of bone cancer pain rats (multiple comparisons of *Tnf*: adjusted *p* < 0.0001, BCP + NC vs. sham + NC; adjusted *p* = 0.0120, BCP + NC vs. BCP + sponge; multiple comparisons of *Il1b*: adjusted *p* < 0.0001, BCP + NC vs. sham + NC; adjusted *p* = 0.0101, BCP + NC vs. BCP + sponge; multiple comparisons of *Il6*: adjusted *p* < 0.0001, BCP + NC vs. sham + NC; adjusted *p* = 0.0177, BCP + NC vs. BCP + sponge; Figure 7D–F), implying the therapeutic effect of inhibiting miR-155-5p, restoring *Sgk3*, and then alleviating microglial activation in bone cancer pain rats.

## 4. Discussion

In this study, we demonstrated that miR-155-5p, which was screened using RNA-seq and confirmed using RT-qPCR, was significantly upregulated and expressed in the cytoplasm of microglia in the spinal cord of bone cancer pain rats. Then, microglial activation with an increase in inflammatory cytokines in bone cancer pain rats was imitated using an LPS treatment in vitro; and miR-155-5p knockdown alleviated the augment of *Tnf*, *Il1b*, and *Il6*, which was possibly achieved by suppressing *Sgk3* in vitro. Finally, inhibiting miR-155-5p in vivo confirmed its regulation of *Sgk3* and activation of microglia, which mediated the BCP development.

The pathogenesis of BCP is complicated and probably involves multiple mechanisms [32] in which aberrantly expressed miRNAs in the CNS are promising core regulatory molecules. Genome-wide miRNA screening combined with in silico analyses is an effective and objective method to find out the aberrantly expressed miRNAs, as Bali, K.K. et al. demonstrated the involvement of the upregulated miR-1a-3p in the dorsal root ganglia (DRG) by inhibiting *Clcn3* expression [33] and miR-34c-5p targeting *Cav2.3* [34]. At the spinal cord level, Wu, X.P. et al. found the protective role of miR-329 through the LPAR1-dependent LPAR1/ERK signal transduction pathway [35]; Elramah S. et al. verified the downregulation of miR-124 in a mouse model of cancer pain, which could inhibit synaptopodin, which is a key protein for synaptic transmission [8]; and Liu, M. showed the downregulation of spinal miR-135-5p in BCP mice, which could inhibit astrocyte-mediated neuroinflammation by blocking the JAK2/STAT3 signaling pathway [9]. However, we did not find the miRNA expression data in the rat spinal cord available online; therefore, we performed RNA-seq and filtered out miR-155-5p as another possible aberrantly expressed miRNA, which was verified using RT-qPCR. In studies on nervous system diseases, miR-155-5p, which is viewed as a pro-inflammatory mediator [36], was involved in Alzheimer’s disease [37], Parkinson’s disease [38], experimental autoimmune encephalomyelitis [39], glioma [40], epilepsy [41], etc. Some researchers also attempted to explore the neuroinflammation mediated by miR-155-5p in neuropathic pain [12,13,14,42,43] or chronic pain due to spinal cord injury [15], but its role in BCP has not been studied so far. In our research, miR-155-5p may have acted as an initiating factor of microglia activation in BCP development, while it is also reasonable that miR-155-5p expression may have been affected by microgliosis as a feedback, which still needs further examination.

In our present study, we found significant microgliosis in the lumbar spinal cord of BCP rats, as represented by the augment of immunofluorescence staining of Iba1 and RT-qPCR of pro-inflammatory cytokines, i.e., *Tnf*, *Il1b*, and *Il6*, secreted by canonical M1 polarized microglia, which is in accordance with some previous studies [22,23,44,45,46]. However, we noticed some results accomplished by Diaz-delCastillo, M. et al. that demonstrated the opposite opinion, namely, that cancer-induced bone pain is completely independent of spinal microglia activity [25,26]. This discrepancy may be explained by the differences in carcinoma strains, animal species, stage of BCP, experiment environment, behavioral stimuli, etc., regarding the technical aspect, and the differences in the interaction between the carcinoma and the host, the immune status of BCP rats, the integrity of the blood-spinal cord barrier, etc., regarding the pathophysiological aspect. These academic devergences in a sense reflect the heterogeneity in the pathogenesis of bone cancer pain, which differs from neuropathic pain and inflammatory pain.

miR-155-5p might be a key regulator in microglial activation in bone cancer pain development, which was confirmed in our in vitro and in vivo study. This mechanism was probably shared by a large spectrum of neuronal disorders, including amyotrophic lateral sclerosis [47], multiple sclerosis [48], Parkinson’s disease [49], and traumatic brain injury [50]. miR-155 is necessary for *Il6* induction in microglia under LPS stimulation in vitro and enhances microglia proliferation and amoeboid morphologic change in vivo [51]. miR-155 could mediate the polarization of microglia from the M2 to the M1 phenotype [42,52].

For the direct target of miR-155-5p, we thought it might be *Sgk3*, which was predicted using bioinformatics, verified using the dual-luciferase reporter system, confirmed on microglia in vitro and BCP rats in vivo, and is consistent with previous studies [31,53]. The widely expressed SGK3 can be activated by phosphatidylinositol 3 kinase (PI3K)/3-phosphoinositide (PIP3)-dependent kinase PDK1 and has a role in neutral amino acid transport and the activation of potassium and chloride channels by phosphorylating several target proteins [54]. Expressed in microglia, the basal level of SGK3 is probably to suppress microglial activation, and its inhibitor promotes microglial inflammatory responses, maybe by enhancing the translocation of NF-κB [55].

We must admit that our understanding of BCP pathogenesis is somewhat superficial, our argument is far from comprehensive, and the experimental design was not perfect due to the condition limitation, such as the species inconsistency between the in vivo and in vitro studies. Further studies are still needed to explore the potential role of miR-155-5p in other neural cells besides microglia in BCP pathogenesis.

## 5. Conclusions

Taken together, this is the first study to explore the role of spinal miR-155-5p in BCP pathogenesis. Our results indicated that tibial carcinoma cell inoculation stimulated the upregulation of spinal miR-155-5p, which inhibited the *Sgk3* level and then promoted microglial activation, finally leading to the development of BCP. Our study provided the novel insight that the miR-155-5p/*Sgk3*/microglia axis could be an important regulatory mechanism in BCP development and might be a promising interventional target for BCP treatment.

## Data Availability

Data are available from the corresponding author upon specific request.

## Supplementary Materials

The following supporting information can be downloaded from https://www.mdpi.com/article/10.3390/life12091349/s1. Figure S1: Lipopolysaccharide treatment at the concentration of 1000 ng/mL of N9 cells induced an increase in miR-155-5p (A) and a dramatic surge of inflammatory factors, i.e., *Tnf*, *Il1b*, and *Il6* (B, C, and D). *n* = 3, ** *p* < 0.01, *** *p* < 0.001 vs. the Con group. Figure S2: Immunofluorescence of SGK3 (red) in the spinal cord showed its co-localization with Iba1 (green, a marker of microglia) and primarily inside of nucleus (marked using DAPI, blue), as indicated by white arrows (scale bar: 50 μm). Table S1: Sequences of primers used in RT-qPCR. Table S2: Statistics of raw reads and clean reads in all samples in the miRNA sequencing assay. Table S3: Statistics of different RNAs in clean reads with 18–40 nt in all samples in the miRNA sequencing assay.
