# Supplementary figures and images for: Upregulation of Spinal miR-155-5p Contributes to Mechanical Hyperalgesia by Promoting Inflammatory Activation of Microglia in Bone Cancer Pain Rats
Source: Life (Basel). 2022 Aug 30;12(9):1349. doi: 10.3390/life12091349 (PMC9503135; doi:10.3390/life12091349)

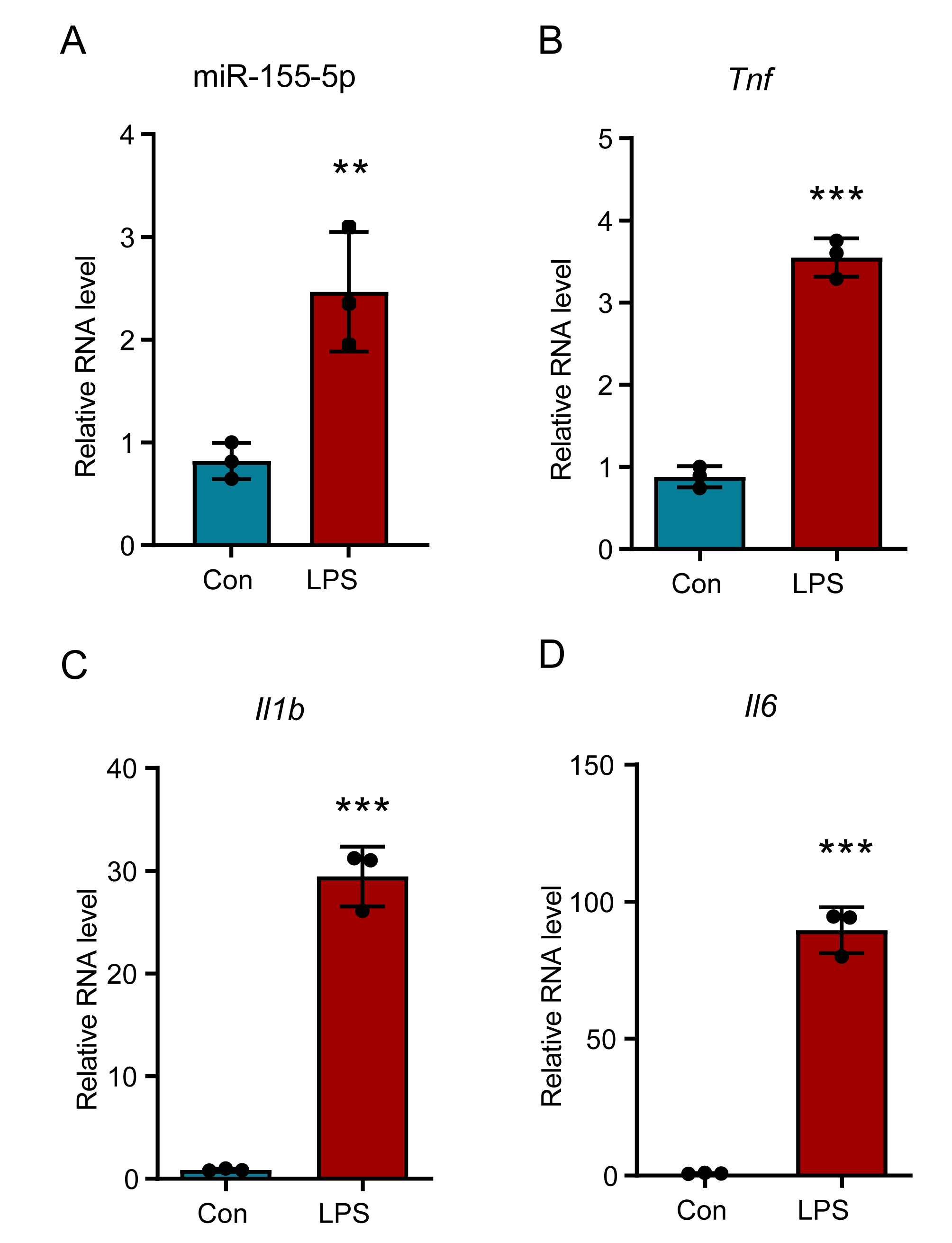

Supplement: Supplementary file 1 [file life-12-01349-s001.zip › Figure S1.tif]

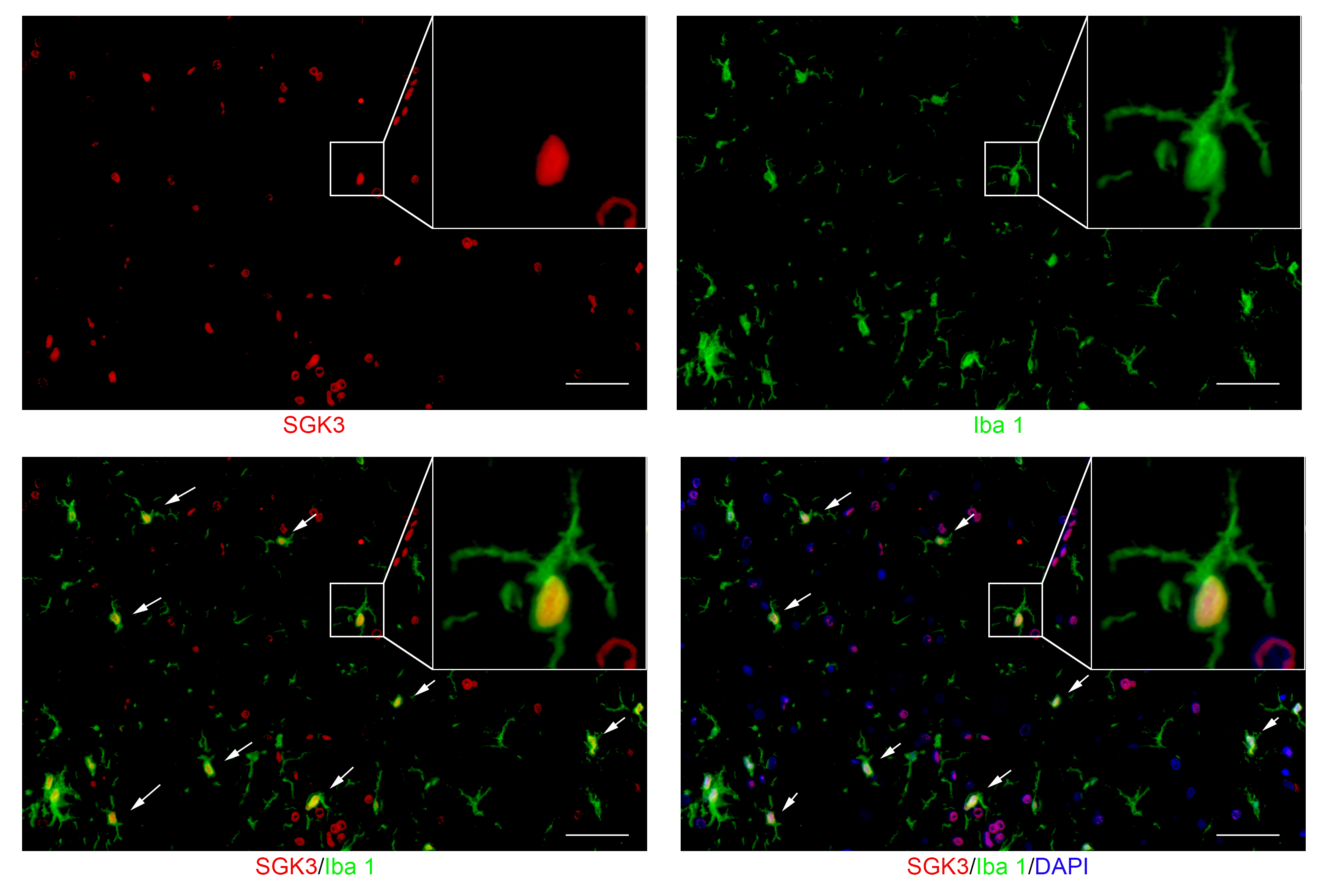

Supplement: Supplementary file 1 [file life-12-01349-s001.zip › Figure S2.tif]
